# Propensity-score matched analysis to evaluate efficacy of endoscopic submucosal dissection for superficial esophageal cancer in gastrectomized patients

**DOI:** 10.1038/s41598-022-15410-4

**Published:** 2022-07-01

**Authors:** Yasuhiko Hamada, Kyosuke Tanaka, Masaki Katsurahara, Noriyuki Horiki, Yuhei Umeda, Yohei Ikenoyama, Hiroki Yukimoto, Masahiko Tameda, Junya Tsuboi, Reiko Yamada, Misaki Nakamura, Hayato Nakagawa

**Affiliations:** Department of Gastroenterology and Hepatology, Mie University Hospital, 2-174 Edobashi, Tsu, Mie 514-8507 Japan

**Keywords:** Gastroenterology, Gastrointestinal diseases

## Abstract

Endoscopic submucosal dissection (ESD) is a minimally invasive treatment option for superficial esophageal cancer (SEC) with high rates of complete resection. However, limited research exists on the efficacy of ESD for SEC in gastrectomized patients. This study aimed to evaluate the efficacy of ESD for SEC in gastrectomized patients. We included 318 patients of SEC treated at our institution between April 2005 and October 2021. To minimize bias between the gastrectomized and non-gastrectomized groups, we conducted a propensity-score matched analysis and compared the ESD outcomes for SEC of the two groups. Of the 318 patients included in the study, 48 and 270 patients were in the gastrectomized and non-gastrectomized groups, respectively. After 1:2 propensity-score matching, we matched 44 patients in the gastrectomized group to 88 patients in the non-gastrectomized group, and found no significant differences in the baseline clinicopathological characteristics. Regarding the ESD outcomes, there were no significant differences in the complete resection rate, procedure time, hospitalized period, and recurrence rates between the two groups. Multivariate analysis also cofirmed that the history of gastrectomy was not a risk factor of the difficult case of esophageal ESD. In conclusion, history of gastrectomy might not negatively affect the ESD outcomes of SECs.

## Introduction

Gastrectomy is a major abdominal surgery for peptic ulcer and gastric cancer worldwide, especially in East Asian countries. Patients who have undergone gastrectomy are at higher risk for esophageal cancer because of duodenogastroesophageal reflux of bile acid^1–4^. In Japan, the incidence of gastrectomy in the general population is 0.87%^5^, and it is significantly higher in patients with esophageal cancer (3.4–10.4%)^5–7^.

Esophagectomy of superficial esophageal cancer (SEC) in gastrectomized patients is technically difficult because the jejunum or colon has to be used instead of the stomach to reconstruct the esophagus. This reconstruction results in a high rate of procedure-related complications^8–10^. Although chemoradiotherapy (CRT) is an alternative to esophagectomy as a treatment option for SEC in gastrectomized patients, a large cohort study revealed that the survival rate in patients with SEC was lower after CRT than after esophagectomy^11^. Therefore, a standard therapeutic strategy is required in gastrectomized patients with SEC.

Endoscopic submucosal dissection (ESD) is a minimally invasive treatment option for SECs and has a curative benefit with rapid recovery and organ preservation^12,13^. Recent reports suggest that the effects of ESD on overall survival are better and the likelihood of disease recurrence is lower than in patients with SEC who underwent esophagectomy^14–16^. Therefore, ESD could be an ideal alternative treatment for SEC in gastrectomized patients, considering the technical difficulties of esophagectomy. However, a previous study showed that the altered anatomy after gastrectomy negatively affected the ESD outcomes of SECs in gastrectomized patients^17^. The study had a single-arm design; therefore, evidence for the ESD outcomes for SECs in gastrectomized patients is still unsatisfactory. This study aimed to evaluate the ESD outcomes of SECs in gastrectomized patients, compared with non-gastrectomized patients, using propensity-score matching.

## Methods

### Study design and patients

We reviewed the medical records of patients with SECs treated by ESD between April 2005 and October 2021 at our institution. We defined SEC as a pathologically confirmed esophageal cancer confined to the submucosa with no lymph node metastasis, diagnosed by computed tomography (CT). A history of gastrectomy was confirmed endoscopically. The study was approved by the local ethics committee of Mie University Hospital (approval number H2021-116) and conducted in accordance with the ethical standards of the Declaration of Helsinki. The local ethics committee approved the opt-out method for obtaining patient consent. The patients provided informed consent on the institutional website.

### ESD procedure

During the study period, various experienced endoscopists performed the ESD. Diazepam and pethidine hydrochloride were used for sedation in all patients during the ESD procedures. All ESDs were performed using an upper gastrointestinal endoscope with a water-jet function (GIF-Q260J; Olympus Medical Systems, Tokyo, Japan). A transparent cap (D-201-11804; Olympus, Tokyo, Japan, or DH-28GR; Fujifilm, Tokyo, Japan) was attached to the tip of the endoscope. We performed electrical cutting and coagulation using a high-frequency electrosurgical unit (VAIO 300D, Erbe Elektromedizin, Tübingen, Germany). A Dualknife (Olympus Medical Systems, Tokyo, Japan) was used as the main electrosurgical knife, and additional electrosurgical knives were used during the procedure depending on the endoscopist’s discretion.

We performed chromoendoscopy by direct instillation of a 1% iodine solution to determine the lateral extent of the lesion. We marked the border of the lesion using dots. We then injected a 10% glycerin solution (Glycerol, Chugai Pharmaceutical Co, Ltd, Tokyo, Japan) with epinephrine (dilution, 1:200,000) into the submucosa around the lesion to lift it, and made an incision on the distal and proximal sides of the lesion. Submucosal dissection was performed from the proximal to the distal area, and the lesion was removed en bloc. When a mucosal defect affecting more than three-quarters of the esophageal circumference occurred after ESD, we injected triamcinolone acetonide (Kenacort; Bristol-Myers Squibb Co., Tokyo, Japan) locally to prevent postoperative esophageal stricture.

The resected specimens were pinned to specimen boards, fixed in formalin, dissected into 2–3 mm-wide slices, and stained with hematoxylin and eosin. According to the Japanese Classification for Esophageal Cancer, we evaluated the specimen size, histologic type, depth of tumor invasion, horizontal and vertical resection margins, and lymphovascular invasion^18^.

### Definitions

We defined an ESD operator who had performed ≥ 30 esophageal ESDs as an expert, and one who had performed < 30 esophageal ESDs as a trainee. We classified tumor location in the esophagus as cervical, upper, middle, lower, or abdominal. Tumor position on the esophagus was classified as posterior, anterior, right, or left wall. The circumferential extent of the tumor on the esophagus was measured as the proportion of the esophageal circumference, calculated by dividing the esophageal lumen into four equal parts (e.g., 1/4 and 3/4). ESD procedure time was defined as the time from submucosal injection to end of resection. En bloc resection was defined as resection of the lesion as a single piece. We defined complete resection as en bloc resection with negative horizontal and vertical margins, and defined curative resection as complete resection with a tumor depth limited to the lamina propria and no lymphovascular invasion according to the ESD guidelines for esophageal cancer in Japan^19^.

Adverse events included postoperative bleeding, esophageal perforation/pneumomediastinum, postoperative pneumonia, and postoperative esophageal stricture. We defined postoperative bleeding as hemorrhage after ESD requiring transfusion or intervention. We diagnosed esophageal perforation at visualization of the mediastinum during ESD and pneumomediastinum at the presence of extraluminal air within the mediastinum on chest CT without confirming perforation during ESD. Postoperative pneumonia was defined as a new or progressive infiltration confirmed on chest radiography or CT. We defined postoperative esophageal stricture as requirement for balloon dilatation. We noted local and distant recurrence during follow-up. Local recurrence was defined as the development of cancer at the site of the previous ESD scar. Distant recurrence was defined as lymphadenopathy or detection of a cancerous lesion in another organ by CT or positron emission tomography. According to a previous report^20^, we defined difficult cases of esophageal ESD as those meeting any of the following criteria: (1) long procedure time (> 120 min), (2) occurrence of perforation/pneumomediastinum, or (3) incomplete resection.

### Salvage treatment after ESD

When resection was curative, we performed endoscopic examination and biopsy of suspicious sites at 2 and 12 months after ESD and 12-month intervals thereafter. When the resection was non-curative (e.g., a positive resection margin, tumor extending into the muscularis mucosa or deeper, or the presence of lymphovascular invasion), we informed the patient about the need for salvage treatment, including surgery, CRT, chemotherapy alone, or radiotherapy alone, and the associated benefits and risks of each. When patients opted to be followed-up without salvage treatment, we performed CT of the neck, chest, and abdomen every 6 months, and endoscopic examination annually.

### Study outcomes

The primary outcome was the complete resection rate and the secondary outcomes were the procedure time, hospitalization period, adverse event rate, and recurrence rate; these outcomes were compared between gastrectomized and non-gastrectomized groups.

### Propensity-score matching

There were confounding differences between the two groups, which might have influenced the esophageal ESD outcomes. Therefore, we carried out propensity-score matching to reduce the confounding bias in each case of the gastrectomized group and non-gastrectomized group. We calculated propensity scores using a logistic regression model. Based on prior knowledge, the following variables were included in the model: age, sex, previous radiotherapy for the esophagus, tumor size, tumor location in the esophagus (upper [cervical/upper thoracic/middle thoracic] esophagus or lower [lower thoracic/abdominal] esophagus), tumor position in the esophagus (anterior/posterior/right wall or left wall), gross type (elevated/flat or depressed), histological type (squamous cell carcinoma or adenocarcinoma), invasion depth (mucosa or submucosa), resection of two or more lesions, and operator’s skill (expert or trainee). After the propensity scores were estimated, we performed a 1:2 nearest neighbor matching using a caliper set at 0.2. Absolute standard differences were used to evaluate the balance of the confounding variables between the two groups after propensity-score matching.

### Statistical analysis

We expressed continuous variables as means (standard deviation [SD]) or median (interquartile range [IQR]) and categorical variables as numbers and frequencies. We used the Student t-test or Mann–Whitney U test to compare continuous variables and the chi-squared test or Fisher’s exact test to compare categorical variables, as appropriate. Risk factors associated with difficult case of esophageal ESD were analyzed using univariate and multivariate analyses with a logistic regression model. Overall, 12 factors were included in the univariate analysis: age, sex, previous radiotherapy for the esophagus, tumor size, tumor location in the esophagus, tumor position in the esophagus, gross type, histological type, invasion depth, resection of two or more lesions, operator’s skill, and the history of gastrectomy. The results of the univariate and multivariate analyses are expressed as odds ratios (ORs) with 95% confidence intervals (CIs). All statistical analyses were performed using the Statistical Package for the Social Sciences version 26 (IBM Corp., Armonk, NY, USA) and EZR version 1.27 (Saitama Medical Center, Jichi Medical University, Japan)^21^. All tests were two-sided, and a *P*-value < 0.05 was considered statistically significant.

### Ethics approval

The study was approved by the ethics committee of Mie University Hospital (approval number H2021-116) and conducted in accordance with the approved protocol and the ethical standards of the Declaration of Helsinki.

### Patient consent

The ethics committee of Mie University Hospital approved the use of an opt-out method to obtain consent; thus, informed consent was obtained via the opt-out option on our facility’s website.

## Results

### Clinicopathological characteristics of all cases

**Figure 1:**
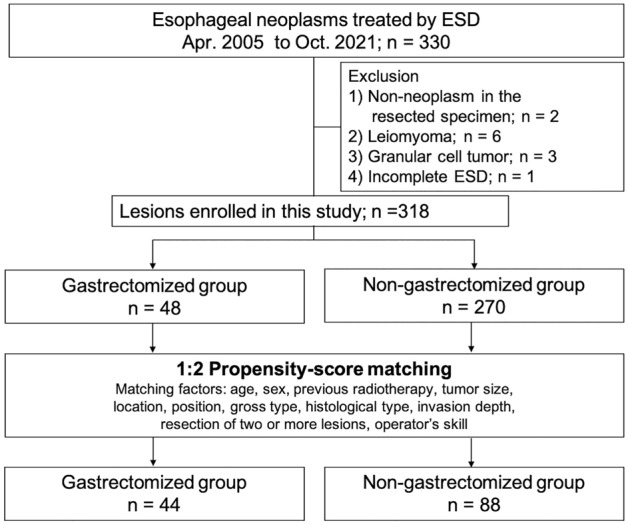
Study flowchart. ESD, endoscopic submucosal dissection.

The study selection process is shown in Fig. 1. In total, 330 cases of esophageal neoplasms were treated by ESD during the study period. Of these neoplasms, 12 cases were excluded; two cases with no evidence of neoplasm in the resected specimen, six cases were leiomyoma, three cases were granular cell tumor, and one case with incomplete ESD. Therefore, 318 cases of SEC were included in the analyses.

Clinicopathological characteristics of the 318 cases are summarized in Table 1. The mean age (SD) was 68.6 (8.7) years, with male predominance (87.1%). The tumors were mainly located in the middle thoracic esophagus (51.3%). Regarding the gross type of the tumor, 61.6% were of the depressed type. The most common histologic type was squamous cell carcinoma (91.2%). Lymphatic invasion and venous invasion were seen in 4.7 and 1.3% cases, respectively.

**Table 1:** Baseline clinicopathological characteristics of all cases treated by endoscopic submucosal dissection (n = 318).

Variable	
Age, mean (SD), years	68.6 (8.7)
**Sex, n (%)**	
Male	277 (87.1)
Female	41 (12.9)
Tumor size, mean (SD), mm	21.9 (13.6)
**Tumor location in the esophagus, n (%)**	
Cervical esophagus	6 (1.9)
Upper thoracic esophagus	24 (7.5)
Middle thoracic esophagus	163 (51.3)
Lower thoracic esophagus	93 (29.2)
Abdominal esophagus	32 (10.1)
**Tumor position in the esophagus, n (%)**	
Anterior wall	56 (17.6)
Posterior wall	98 (30.8)
Right wall	105 (33.0)
Left wall	59 (18.6)
**Gross type, n (%)**	
Elevated	35 (11.0)
Flat	87 (27.4)
Depressed	196 (61.6)
**Histological type, n (%)**	
Squamous cell carcinoma	290 (91.2)
Adenocarcinoma	28 (8.8)
**Invasion depth, n (%)**	
Mucosa	288 (90.6)
Submucosa	30 (9.4)
Lymphatic invasion positive, n (%)	15 (4.7)
Venous invasion positive, n (%)	4 (1.3)

*SD* Standard deviation.

### Clinicopathological characteristics before and after propensity-score matching

Comparisons of the clinicopathologic characteristics between the non-gastrectomized and gastrectomized groups before and after propensity-score matching are shown in Table 2. Before propensity-score matching, there were 48 cases in the gastrectomized group and 270 cases in the non-gastrectomized group. There was a difference in previous radiotherapy for the esophagus, tumor location, and histological type; however, these findings were not statistically significant (previous radiotherapy for the esophagus, *P* = 0.050; tumor location, *P* = 0.077; histological type, *P* = 0.096).

**Table 2:** Baseline clinicopathological characteristics of the non-gastrectomized and gastrectomized groups before and after propensity-score matching.

Variable	All cases (n = 318)				Propensity-score matched cases (n = 132)			
	Non-gastrectomized group (n = 270)	Gastrectomized group (n = 48)	ASD	*P-*value	Non-gastrectomized group (n = 88)	Gastrectomized group (n = 44)	ASD	*P-*value
Age, mean (SD), years	68.7 (9.0)	68.3 (7.1)	0.045	0.791	70.1 (8.3)	68.9 (6.7)	0.034	1.000
Sex, male, n (%)	237 (87.8)	40 (83.3)	0.127	0.360	77 (87.5)	38 (86.4)	0.169	0.404
Previous radiotherapy for the esophagus, n (%)	20 (7.4)	8 (16.7)	0.287	0.050	12 (13.6)	6 (13.6)	< 0.001	1.000
Previous esophagectomy, n (%)	7 (2.6)	0 (0.0)	0.231	0.600	2 (2.3)	0 (0.0)	0.216	0.552
Tumor size, mean (SD), mm	22.1 (13.7)	20.5 (13.2)	0.117	0.461	21.9 (14.5)	20.4 (13.3)	0.026	0.886
Circumferential extent of the tumor > 1/2, n (%)	97 (35.9)	18 (37.5)	0.033	0.871	35 (39.8)	18 (40.9)	0.023	1.000
**Tumor location in the esophagus, n (%)**			0.307	0.077			0.049	0.844
Upper (cervical/upper thoracic/middle thoracic)	158 (58.5)	35 (72.9)			60 (68.2)	31 (70.5)		
Lower (lower thoracic/abdominal)	112 (41.5)	13 (27.1)			28 (31.8)	13 (29.5)		
**Tumor position in the esophagus, n (%)**			0.128	0.421			< 0.001	1.000
Anterior/posterior/right wall	222 (82.2)	37 (77.1)			70 (79.5)	35 (79.5)		
Left wall	48 (17.8)	11 (22.9)			18 (20.5)	9 (20.5)		
Gross type, n (%)			0.284	0.107			0.025	1.000
Elevated/flat	109 (40.4)	13 (27.1)			27 (30.7)	13 (29.5)		
Depressed	161 (59.6)	35 (72.9)			61 (69.3)	31 (70.5)		
**Histological type, n (%)**			0.337	0.096			< 0.001	1.000
Squamous cell carcinoma	243 (90.0)	47 (97.9)			86 (97.7)	43 (97.7)		
Adenocarcinoma	27 (10.0)	1 (2.1)			1 (2.3)	1 (2.3)		
**Invasion depth, n (%)**			0.138	0.593			0.047	1.000
Mucosa	243 (90.0)	45 (93.8)			83 (94.3)	41 (93.2)		
Submucosa	27 (10.0)	3 (6.2)			3 (6.8)	3 (6.8)		
Lymphatic invasion positive, n (%)	14 (5.2)	1 (2.1)	0.166	0.709	6 (6.8)	1 (2.3)	0.220	0.423
Venous invasion positive, n (%)	4 (1.5)	0 (0.0)	0.173	1.000	1 (1.1)	0 (0.0)	0.152	1.000
Resection of two or more lesions, n (%)	40 (14.8)	9 (18.8)	0.105	0.515	18 (20.5)	9 (20.5)	< 0.001	1.000
**Operator’s skill, n (%)**			0.080	0.564			0.186	0.401
Expert	217 (80.4)	37 (77.1)			63 (71.6)	35 (79.5)		
Trainee	53 (19.6)	11 (22.9)			25 (28.4)	9 (20.5)		

*ASD* Absolute standardized difference, *SD* Standard deviation.

After propensity score matching, we matched 44 cases in the gastrectomized group to 88 cases in the non-gastrectomized group (Table 2). There were no significant differences in any of the clinicopathological characteristics between the two groups. The matching of the two groups was balanced, with the absolute standardized differences of all factors within ± 1.96√2/n^22^.

### ESD outcomes after propensity-score matching

The ESD outcomes after propensity-score matching were compared between the two groups (Table 3). After propensity-score matching, the en bloc resection and complete resection rates between the two groups were the same. Although statistical significance was not reached (*P* = 0.178), the procedure time of non-gastrectomized group tended to be longer than that of gastrectomized group (gastrectomized group, 75.4 min; non-gastrectomized group, 92.3 min). Neither the adverse event rate nor the recurrence rate showed a significant difference between the two groups. No significant difference in cases meeting the criteria of difficult ESD was noted.

**Table 3:** ESD outcome comparisons between the non-gastrectomized and gastrectomized groups after propensity-score matching.

Variable	Non-gastrectomized group (n = 88)	Gastrectomized group (n = 44)	*P-*value
**Procedure characteristics**			
Use of two or more electrosurgical knives, n (%)	34 (38.6)	15 (34.1)	0.703
Use of traction device, n (%)	16 (18.2)	8 (18.2)	1.000
Procedure time, mean (SD), min	92.3 (74.0)	75.4 (52.0)	0.178
Procedure time > 120 min	18 (20.5)	7 (15.9)	0.530
Hospitalized period, mean (SD), days	7.0 (5.4)	7.1 (1.9)	0.924
**Resection type**			
En block resection, n (%)	88 (100)	44 (100)	–
Complete resection, n (%)	78 (88.6)	39 (88.6)	1.000
Curative resection, n (%)	60 (68.2)	32 (72.7)	0.689
**Adverse event**			
Postoperative bleeding, n (%)	0 (0.0)	0 (0.0)	–
Esophageal perforation/pneumomediastinum, n (%)	2 (2.3)	1 (2.3)	1.000
Postoperative pneumonia, n (%)	2 (2.3)	0 (0.0)	0.552
Postoperative esophageal stricture, n (%)	8 (9.1)	2 (4.5)	0.495
Difficult ESD cases, n (%)	26 (29.5)	12 (27.3)	0.786
Salvage treatment, n (%)	7 (8.0)	0 (0.0)	0.095
Follow up period, median (IQR), months	37.2 (51.6)	43.1 (44.4)	0.772
**Recurrence**			
Local recurrence, n (%)	0 (0.0)	0 (0.0)	–
Distant recurrence, n (%)	0 (0.0)	0 (0.0)	–

*ESD* Endoscopic submucosal dissection, *SD* Standard deviation, *IQR* Interquartile range.

### Logistic regression analyses of difficult case of esophageal ESD

To analyze the risk factors associated with the difficult case of esophageal ESD, univariate and multivariate regression analyses were conducted (Table 4). The univariate analysis showed that the tumor size and resection of two or more lesions were related to the difficult cases of esophageal ESD. A further multivariate analysis confirmed that tumor size (OR, 1.182; 95% CI, 1.106–1.263; *P* < 0.001) was an independent risk factor for the difficult cases of esophageal ESD. Nevertheless, the history of gastrectomy was not a risk factor for the difficult cases of esophageal ESD in these analyses.

**Table 4:** Univariate and multivariate analyses of risk factors for difficult case of esophageal endoscopic submucosal dissection.

Factor	Univariate analysis		Multivariate analysis	
	Odds ratio (95% CI)	*P-*value	Odds ratio (95% CI)	*P-*value
Age, years	0.969 (0.914–1.013)	0.141	0.942 (0.872–1.018)	0.133
**Sex**				
Male	1		1	
Female	1.897 (0.664–5.421)	0.232	3.341 (0.704–15.860)	0.129
**History of gastrectomy, n (%)**				
Absence	1		1	
Presence	0.894 (0.399–2.002)	0.786	1.131 (0.366–3.499)	0.830
**Previous radiotherapy for the esophagus, n (%)**				
Absence	1		1	
Presence	1.704 (0.606–4.789)	0.312	2.796 (0.611–12.807)	0.185
Tumor size, mean (SD), mm	1.153 (1.096–1.213)	< 0.001	1.182 (1.106–1.263)	< 0.001
**Tumor location in the esophagus, n (%)**				
Upper (cervical/upper thoracic/middle thoracic)	1		1	
Lower (lower thoracic/abdominal)	1.706 (0.733–3.766)	0.186	2.491 (0.685–9.052)	0.166
**Tumor position, n (%)**				
Anterior/posterior/right wall	1		1	
Left wall	1.618 (0.662–3.950)	0.291	1.441 (0.402–5.165)	0.575
**Gross type, n (%)**				
Elevated/flat	1		1	
Depressed	0.110 (0.479–2.503)	0.829	1.493 (0.395–5.646)	0.555
**Histological type, n (%)**				
Squamous cell carcinoma	1		1	
Adenocarcinoma	1.243 (0.109–1.430)	0.861	2.420 (0.156–37.508)	0.527
**Invasion depth, n (%)**				
Mucosa	1		1	
Submucosa	2.647 (0.627–11.183)	0.185	1.672 (0.126–22.226)	0.697
**Resection of two or more lesions, n (%)**				
Absence	1		1	
Presence	0.250 (0.070–0.888)	0.032	0.299 (0.038–2.335)	0.250
**Operator’s skill, n (%)**				
Expert	1		1	
Trainee	0.697 (0.283–1.718)	0.433	0.624 (0.181–2.146)	0.454

*CI* Confidence interval, *SD* Standard deviation.

### Subset analysis

Details of previous gastric surgery in the gastrectomized group after matching are shown in Table 5. Of the 44 cases, distal gastrectomy was performed in 37 cases (84.1%), and total gastrectomy was performed in seven cases (15.9%). The ESD outcomes according to the operation method in the matched gastrectomized group are summarized in Table 6. There were no significant differences in the ESD outcomes between the two groups.

**Table 5:** Details of previous gastric surgery in the gastrectomized group after propensity-score matching (n = 44).

**Indication for gastrectomy, n (%)**	
Malignant disease	17 (38.6)
Benign disease	27 (61.4)
**Operation method, n (%)**	
Total gastrectomy	7 (15.9)
Distal gastrectomy	37 (84.1)
**Reconstruction methods, n (%)**	
Billroth I	22 (50.0)
Billroth II	10 (22.7)
Roux-en-Y	12 (27.3)

**Table 6:** Subset analysis of the ESD outcomes according to the operation methods of gastrectomy after propensity-score matching.

Variable	Distal gastrectomy group (n = 37)	Total gastrectomy group (n = 7)	*P-*value
**Procedure characteristics**			
Use of two or more electrosurgical knives, n (%)	13 (35.1)	2 (26.8)	1.000
Use of traction device, n (%)	7 (18.9)	1 (12.5)	1.000
Procedure time, mean (SD), min	73.9 (53.0)	83.4 (48.7)	0.662
Procedure time > 120 min, n (%)	5 (13.5)	2 (28.6)	0.307
Hospitalized period, mean (SD), days	7.0 (1.9)	7.4 (1.9)	0.614
**Resection type**			
En block resection, n (%)	37 (100.0)	7 (100.0)	–
Complete resection, n (%)	32 (87.2)	7 (100.0)	0.574
Curative resection, n (%)	26 (70.3)	6 (85.7)	0.653
**Adverse event**			
Postoperative bleeding, n (%)	0 (0.0)	0 (0.0)	–
Esophageal perforation/pneumomediastinum, n (%)	1 (2.7)	0 (0.0)	1.000
Postoperative pneumonia, n (%)	0 (0.0)	0 (0.0)	–
Postoperative esophageal stricture, n (%)	1 (2.7)	1 (14.3)	0.296
Difficult ESD cases	10 (27.0)	2 (28.6)	1.000
Salvage treatment, n (%)	0 (0.0)	0 (0.0)	–
Follow up period, median (IQR), months	38.8 (45.5)	46.9 (33.1)	0.975
**Recurrence**			
Local recurrence, n (%)	0 (0.0)	0 (0.0)	–
Distant recurrence, n (%)	0 (0.0)	0 (0.0)	–

*ESD* Endoscopic submucosal dissection, *SD* Standard deviation, *IQR* Interquartile range.

## Discussion

This was the study that compared ESD outcomes for SECs in gastrectomized and non-gastrectomized cases. We analyzed 318 cases (48 and 270 cases in the gastrectomized and non-gastrectomized groups, respectively) in this study. After 1:2 matching according to propensity-score matching, 44 cases in the gastrectomized group were matched to 88 cases in the non-gastrectomized group. The clinicopathological characteristics of the matched cases were balanced between the two groups. In terms of the ESD outcomes, no significant differences were found between the two groups. The multivariate analysis also confirmed that the history of gastrectomy was not a risk factor of the difficult case of esophageal ESD.

A history of gastrectomy is considered an important factor associated with the management of SECs. Esophagectomy is technically difficult for SECs developed after gastrectomy because the remaining stomach is not suitable for esophageal reconstruction^8,10^. Although another alternative treatment for these cases is CRT, failure was observed in 32–60% of the patients with SEC receiving CRT^23–25^. Under these circumstances, given the efficacy of the ESD for SECs in previous reports, we assume that ESD is another option for the treatment of SEC developed after gastrectomy.

Only a single-armed study has been conducted to evaluate the efficacy of ESD for SEC developing after gastrectomy^17^; therefore, it is still controversial whether a history of gastrectomy affects the ESD outcomes for SECs. Furthermore, the propensity-score matched analysis is a statistical technique that addresses confounding bias and mimics a randomized clinical trial, improving the level of evidence in studies^26,27^. To avoid the confusing relationship between a history of gastrectomy and the ESD outcomes for SECs, we carried out propensity-score matching to balance the baseline clinicopathological characteristics.

A previous report showed that ESD for SEC in gastrectomized patients was associated with an en bloc resection rate and complete resection rate of 94.6 and 86.5%, respectively^17^. These rates were lower than those reported previously in non-gastrectomized patients, who had en bloc resection rates of nearly 100% and complete resection rates of 87.9–97.4%^13,28,29^. The authors discussed that this might be due to the altered anatomy after gastrectomy, which impaired the resectability of ESD^17^.

Conversely, in our study, the en bloc and complete resection rates in the matched gastrectomized group were 100 and 88.6%, respectively, and were not significantly different from those in the matched non-gastrectomized group. These results are similar to those of previous studies on SECs treated by ESD in patients without a history of gastrectomy^13,28,29^. Moreover, other ESD outcomes were not significantly different from those in the non-gastrectomized group, and were similar to those in previous studies that included patients without a history of gastrectomy^28–30^. Therefore, our findings confirm that the ESD outcomes for SECs in gastrectomized patients were not inferior to their non-gastrectomized counterparts.

This study had several strengths. First, to our knowledge, this is the first study with a double-armed design to investigate the ESD outcomes for SEC in gastrectomized and non-gastrectomized patients. A previous study addressed the efficacy of ESD for SEC in gastrectomized patients, but only included a single-arm^17^. Therefore, our study provided more substantial evidence regarding the ESD outcomes for SECs in gastrectomized patients. Second, compared with previous studies, a larger number of endoscopists with varying skill levels participated in this study. Therefore, our conclusions may be more generalizable than those of previous studies. Finally, to minimize the selection bias caused by the baseline clinicopathological characteristics in each group, we conducted propensity-score matched analyses and found that a history of gastrectomy did not negatively affect the ESD outcomes.

Our study also had several limitations. First, it was not a randomized, controlled study, although propensity-score matching was performed to reduce biases between the two groups. Second, heterogeneity of the operators and different timelines in each group may have led to bias. Third, the procedure time of non-gastrectomized group tended to be longer than that of gastrectomized group. We think that the reason could be the proportion of trainees in the non-gastrectomized group was higher than that in the gastrectomized group, although statistical significance was not reached (*P* = 0.401). In contrast, the reason why the complete resection rates were almost similar in the two groups is that the trainee was always accompanied by an expert as an education operator, when ESD was performed. Accordingly, the higher proportion of trainees in the non-gastrectomy group than that in the gastrectomy group is one of the limitations in comparing the ESD outcomes in this study. Finally, to decrease the selection bias of baseline clinicopathological characteristics, we conducted a propensity-score matched analysis; however, the results were based on a small sample size that might have been underpowered to detect significant differences. Therefore, a definite conclusion needs more substantial evidence, and future studies with larger sample sizes are required to evaluate the efficacy of ESD for SECs in gastrectomized patients.

In conclusion, a history of gastrectomy may not affect the ESD outcomes of SECs negatively. ESD is considered an effective and feasible treatment for SECs not only in non-gastrectomized patients, but also in gastrectomized patients.

## Data Availability

The datasets generated during and/or analysed during the current study are available from the corresponding author on reasonable request.
